# Poster Session I - A163 A COLORECTAL CANCER-ASSOCIATED FECAL MIRNOME AND ITS POTENTIAL TO ALTER *FUSOBACTERIUM NUCLEATUM* AND *ESCHERICHIA COLI* GENE EXPRESSION

**DOI:** 10.1093/jcag/gwaf042.163

**Published:** 2026-02-13

**Authors:** J Pan, L Colvile, E M Comelli

**Affiliations:** University of Toronto Department of Nutritional Sciences, Toronto, ON, Canada; University of Toronto Department of Nutritional Sciences, Toronto, ON, Canada; University of Toronto Department of Nutritional Sciences, Toronto, ON, Canada

## Abstract

**Background:**

Intestinal cells release microRNAs (miRNAs) in the lumen, which contribute to the fecal miRNome. We found that this contains a core of miRNAs shared across healthy individuals. Select fecal miRNAs have been proposed as biomarkers of cancers, including colorectal cancer (CRC). However, the existence of a shared fecal miRNome in CRC is unknown. This is important because beyond serving as biomarkers, fecal miRNAs were suggested as modulators of bacterial growth in the gut. Gut microbiome alteration is linked with CRC transformation and progression. A subset of *Fusobacterium nucleatum* and *Escherichia coli* strains contributes to CRC dysbiosis and has been linked to carcinogenesis in the gut. However, the interaction between CRC fecal miRNAs and these bacteria remains under-investigated.

**Aims:**

To determine the shared fecal miRNome in patients with CRC and to identify the potential gene targets of shared miRNAs in *F. nucleatum* and *E. coli* strains.

**Methods:**

PubMed and the Cochrane library were systematically searched for fecal miRNA profiling studies on patients with CRC. Shared miRNA species across the extracted datasets were identified (ComplexHeatmap R package). The genomes of select *F. nucleatum* and *E. coli* strains were extracted from NCBI and used to identify potential gene targets of the shared miRNAs via BLAST, followed by in silico validation (RNAup, ViennaRNA software).

**Results:**

Of the 157 papers identified, 154 were excluded (2 due to duplication, 39 during title and abstract screening, 113 during full text review); 3 datasets were extracted from the 3 remaining papers (N = 298). 31 miRNAs were found to be present in all 3 datasets. Both shared and unique genes targets were found across *F. nucleatum* strains (6-11 targets/strain), which differed from those found in *E. coli* (13 targets). Many of these genes are known to be involved in key cellular activities such as translation, purine/vitamin metabolisms, and metabolite transportation.

**Conclusions:**

Fecal miRNomes of patients with CRC exhibit some degrees of similarity, which may signify the existence of a core fecal miRNA signature in CRC, potentially a marker of disease progression and gut microbiota dysbiosis. *F. nucleatum* and *E. coli* gene expression can potentially be modulated by miRNAs found in CRC stools in a different manner, which may serve as a novel approach to understand host-microbe interaction in the context of CRC.

**Funding Agencies:**

NSERC, Canada Graduate Scholarship-Master’s (CGS M) - CIHR

